# Identification of Palmitoyltransferase and Thioesterase Enzymes That Control the Subcellular Localization of Axon Survival Factor Nicotinamide Mononucleotide Adenylyltransferase 2 (NMNAT2)*

**DOI:** 10.1074/jbc.M114.582338

**Published:** 2014-09-30

**Authors:** Stefan Milde, Michael P. Coleman

**Affiliations:** From the Babraham Institute, Babraham Research Campus, Cambridge CB22 3AT, United Kingdom

**Keywords:** Enzyme, Intracellular Trafficking, Neurodegeneration, Protein Acylation, Protein Palmitoylation, APT1, APT2, NMNAT2, Palmitoyltransferase, zDHHC17

## Abstract

The NAD-synthesizing enzyme nicotinamide mononucleotide adenylyltransferase 2 (NMNAT2) is a critical survival factor for axons and its constant supply from neuronal cell bodies into axons is required for axon survival in primary culture neurites and axon extension *in vivo*. Recently, we showed that palmitoylation is necessary to target NMNAT2 to post-Golgi vesicles, thereby influencing its protein turnover and axon protective capacity. Here we find that NMNAT2 is a substrate for cytosolic thioesterases APT1 and APT2 and that palmitoylation/depalmitoylation dynamics are on a time scale similar to its short half-life. Interestingly, however, depalmitoylation does not release NMNAT2 from membranes. The mechanism of palmitoylation-independent membrane attachment appears to be mediated by the same minimal domain required for palmitoylation itself. Furthermore, we identify several zDHHC palmitoyltransferases that influence NMNAT2 palmitoylation and subcellular localization, among which a role for zDHHC17 (HIP14) in neuronal NMNAT2 palmitoylation is best supported by our data. These findings shed light on the enzymatic regulation of NMNAT2 palmitoylation and highlight individual thioesterases and palmitoyltransferases as potential targets to modulate NMNAT2-dependent axon survival.

## Teaser

**Background:** Axon survival factor nicotinamide mononucleotide adenylyltransferase 2 (NMNAT2) is targeted to Golgi membranes through palmitoylation, resulting in its rapid turnover and axon degeneration.

**Results:** Thioesterases APT1/APT2 depalmitoylate NMNAT2 and zDHHC17 is the strongest candidate palmitoyltransferase for NMNAT2.

**Conclusion:** Depalmitoylation is insufficient to release NMNAT2 from membranes, suggesting palmitoylation-independent mechanisms contribute to membrane association.

**Significance:** Modulation of NMNAT2 subcellular localization could delay axon degeneration in neurodegenerative disease.

## Introduction

The NAD-synthesizing enzyme nicotinamide mononucleotide adenylyltransferase 2 (NMNAT2)^2^ is critically required for axon survival in primary culture neurons (1) and axon growth and maintenance *in vivo* (2). As a very short-lived protein that is transported into axons from the cell body, the half-life of NMNAT2 limits the duration of survival of a distal axon stump after its physical separation from the cell body (1). Additionally, a drop in the supply of NMNAT2 from neuronal cell bodies into axons could contribute to axon degeneration in neurodegenerative diseases, where axonal transport has been shown to be impaired at an early stage (3). In support of this, expression of a more stable, axonally targeted NMNAT enzyme can rescue axon degeneration and phenotypic disease onset in animal models of several neurodegenerative conditions including motoneuron disease (4), Parkinson disease (5, 6), glaucoma (7), and myelin-related axon loss (8).

Palmitoylation of a central double-cysteine motif is required for the association of NMNAT2 with Golgi membranes in HeLa cells (9, 10) and with axonal transport vesicles in primary culture neurons (11). In a previous study we found that a small, central region within the isoform-specific targeting and interaction domain (cISTID) of NMNAT2, which contains the palmitoylated cysteine residues, is sufficient for successful targeting to Golgi membranes and for axonal transport in primary culture neurons (11). In addition to the palmitoylated cysteines themselves, a number of basic amino acid residues within the cISTID region are also required for efficient palmitoylation and membrane targeting as we found that in their absence (NMNAT2ΔBR) palmitoylation and membrane targeting are greatly reduced (11). Furthermore, the palmitoylated cISTID region is also necessary for association of fluorescently tagged NMNAT2 with axonal transport vesicles in mouse peripheral axons *in vivo* (12). Previously, we reported that its subcellular localization alters NMNAT2 stability, with membrane binding resulting in faster turnover due to higher levels of ubiquitination. In contrast, palmitoylation-deficient, cytosolic mutant NMNAT2 was more stable and, as a result of its increased half-life, delayed axon degeneration after cut in mouse primary culture neurites and *Drosophila* axons *in vivo* significantly more strongly than wild-type NMNAT2 (11, 12). As palmitoylation appears to control the subcellular localization of NMNAT2, it is important to identify the enzymes and mechanisms that regulate NMNAT2 palmitoylation and membrane association.

Palmitoylation is the reversible attachment of palmitic acid groups to specific cysteine residues in target proteins via thioesterase linkages. As a dynamic, post-translational fatty acid modification it regulates membrane interactions, subcellular targeting, activity, and turnover of a wide variety of target proteins (13, 14). In neurons specifically, the palmitoylation-dependent subcellular targeting and trafficking of substrate proteins is critical for neuronal function (15–17). The large zDHHC family of palmitoyltransferases mediates the palmitoylation of several target proteins, with varying degrees of substrate specificity (18, 19). In contrast, few enzymes are known to carry out enzymatic depalmitoylation reactions. These include palmitoyl-protein thioesterase 1 and 2, enzymes thought to localize to lysosomes and mediate depalmitoylation of substrate proteins prior to lysosomal degradation, and the cytosolic thioesterases acyl-protein thioesterase 1 and 2 (APT1 and APT2), which have been shown to depalmitoylate several substrates (20–22) and play roles in palmitate cycling on some target proteins (23, 24).

Here, we report that thioesterases APT1 and APT2 are both able to depalmitoylate NMNAT2. Interestingly, however, there appears to be dissociation between NMNAT2 depalmitoylation and membrane detachment, suggesting alternate mechanisms that mediate stable anchoring of NMNAT2 at membranes. Within NMNAT2, the cISTID region appears to be sufficient for this stable, post-palmitoylation membrane attachment. Additionally, we identify a subset of zDHHC palmitoyltransferases that are capable of mediating NMNAT2 palmitoylation and membrane targeting. Among these, zDHHC17 emerges as the strongest candidate for an endogenous role in regulating levels of NMNAT2 palmitoylation and its subcellular localization.

## EXPERIMENTAL PROCEDURES

### 

#### 

##### DNA Constructs

Mouse zDHHC-HA constructs were a gift from Luke Chamberlain (Glasgow, UK) with kind permission from Masaki Fukata (Okazaki, Japan). Note that throughout this paper, palmitoyltransferases are referred to according to the new standard *zDHHC* nomenclature. For creation of EGFP-tagged *zDHHC* constructs, relevant coding sequences were transferred to the pEGFP-NI vector (Clontech). Fusions of wild-type and variant *Nmnat2* with FLAG, EGFP, and photoactivatable GFP (PA-GFP) tags (11) as well as FLAG-*Wld^S^* and FLAG-*Nmnat1* (1) were described previously. Note that *cISTID*-PA-GFP and FLAG-*Nmnat2*Δ*cISTID* constructs were previously referred to as *Exon6*-PA-GFP and FLAG-*Nmnat2*Δ*ex6*, respectively (11). Table 1 provides an overview of all NMNAT2 mutants used in this study. FLAG-*APT1* and FLAG-*APT2* constructs were generated by PCR amplification of the *APT1* (NM_008866.2) and *APT2* (NM_011942.1) coding sequences from a mouse cDNA library and insertion into pCMV-Tag2A vector (Stratagene). Enzymatically inactive *zDHHC7* (C160S referred to as zDHHS7), *zDHHC17* (C467S; zDHHS17), *APT1* (S119A), and *APT2* (S122A) constructs were created using the QuikChange site-directed mutagenesis kit (Stratagene) according to the manufacturer's instructions.

**TABLE 1:** **NMNAT2 mutants used in this study**

Construct	Description	Mutations
NMNAT2ΔPS	Mutation of palmitoylation site within the cISTID	C164S, C165S
NMNAT2ΔBR	Mutation of all basic residues within the cISTID domain	K151A, K155A, R162A, R167A, R172A
cISTID	Central portion of the ISTID domain (amino acids 150–177 of full-length NMNAT2)	NA*^a^*
NMNAT2ΔcISTID	Deletion of the central portion of the ISTID domain	Δ151–177

*^a^* NA, non applicable.

##### Animals

All animal work was carried out in accordance with the Animals (Scientific Procedures) Act, 1986, under Project Licenses 80/2254 and 70/7620. C57BL/6JBabr mice (BSU, Babraham Institute) were used as a source of wild-type material.

##### Primary Neuronal Culture and Photoactivation Imaging

Dissociated superior cervical ganglia (SCG) cultures were prepared and maintained, and DNA microinjection was carried out as described previously (11). For photoactivation experiments, 0.03 μg/μl of *Nmnat2*-PA-GFP (or variant construct) were co-injected with 0.01 μg/μl of mCherry expression vector and 0.03 μg/μl of either FLAG-*Nmnat1* (control), a *zDHHC*-HA expression construct, FLAG-*APT1* or FLAG-*APT2* (where both 0.02 μg/μl of FLAG-APT1 and FLAG-APT2 were co-injected). Twenty-four hours after microinjection, photoactivation imaging was carried out on an Andor Spinning Disk Confocal System (Nikon 2000E microscope, 100 × 1.40 NA plan apochromat objective, 488 and 561 nm laser excitation). Where indicated, 50 μm palmostatin B (PSB; Calbiochem) or 50 mm hydroxylamine (HA^2^; Sigma) were added 30 or 60 min prior to commencement of imaging (see text). The use of a photoactivatable fluorescent protein fusion to study the extent of NMNAT2 membrane association was described previously (11). Briefly, microinjected cell bodies were identified based on their mCherry fluorescence. PA-GFP fluorescence was then activated by 10 pulses (20-μs pixel dwell time) of a 405 nm laser at 5% intensity in a small 30 × 30 pixel region of interest in the cell body. The pool of photoactivated protein was then followed by taking subsequent images at 1 frame/s (200-ms exposure time) for 3 min. Analysis of protein mobility was then carried out as described (11).

##### Co-migration Analysis

Dissociated SCG neurons were microinjected with 0.03 μg/μl each of *Nmnat2-*mCherry and the indicated *zDHHC-*EGFP construct. Twenty-four hours after injection, time lapse imaging of axonal transport and analysis of co-migration were performed as described previously (11).

##### Cell Culture and Palmitate Labeling

HEK 293 cells were maintained in culture, transfected and labeled with [9,10-^3^H]palmitate (PerkinElmer Life Sciences) as described (11). Where indicated, 50 μm PSB was added at the same time as the radiolabeled palmitate or 5 h after addition of palmitate (*i.e.* 1 h before cell lysis). HA (50 mm) was added for 60 min prior to cell lysis (*i.e.* 5 h after addition of radiolabel). Neuroblastoma X spinal cord cells (NSC34) were maintained in DMEM (Invitrogen) supplemented with 10% FBS (Sigma), 1% penicillin/streptomycin (Invitrogen), 2 mm l-glutamine (Invitrogen), and 1 mm sodium pyruvate (Invitrogen). NSC34 cells in six-well dishes were transfected with 4 μg of FLAG-*Nmnat2* and 1.35 μg of each of the indicated mouse siRNA construct(s) (Thermo Scientific) using Lipofectamine 2000 (Invitrogen) according to the manufacturer's instructions. Seventy-two hours after transfection, palmitate labeling was carried out as for HEK 293 cells.

##### Emetine Chase and Ubiquitination Studies

HEK 293 cells in 24-well dishes were transfected with FLAG-*Wld^S^* and FLAG*-Nmnat2* and subjected to an emetine chase experiment as described previously (11). Where indicated, 50 μm PSB was added 12 h prior to commencement of the emetine chase. Pulldown of ubiquitinated FLAG-NMNAT2 using wild-type or mutant GST-Dsk2 UBA constructs was performed as described (25, 26).

##### Western Blotting

SDS-PAGE and Western blotting analysis were performed as described (1). The following antibody concentrations were used: mouse monoclonal anti-FLAG (Sigma, M2), 1:3,000; mouse monoclonal anti-GFP (Roche Applied Science), 0.1 μg/ml; and Alexa Fluor680-conjugated anti-mouse secondary antibody (Molecular Probes), 1:5,000. Blots were scanned and quantified using the Odyssey imaging system (LI-COR Biosciences).

##### Co-immunoprecipitation

For co-immunoprecipitation of NMNAT2 and zDHHC palmitoyltransferases, HEK 293 cells grown in 6-well dishes were co-transfected with equal amounts of FLAG-*Nmnat2* (or, where indicated, FLAG-*Nmnat2*Δ*cISTID*) and the relevant *zDHHC-*EGFP constructs. 24 h after transfection, cells were washed once in PBS and lysed in 500 μl of TG lysis buffer (20 mm Tris, pH 7.5, 137 mm NaCl, 1 mm EGTA, 1% Triton X-100, 10% glycerol, 1.5 mm MgCl_2_, 50 mm NaF, 1 mm Na_3_VO_4_ and protease inhibitor mixture). After a pre-clearing spin (10 min, 13,000 rpm, 4 °C), 5 μg of anti-FLAG (Sigma, M2) or anti-GFP (Roche) antibody was added to the supernatant and mixed overnight at 4 °C. The following day, 50 μl of 50% washed Protein-G Sepharose beads (Sigma) were added and the sample was mixed for 3 h at 4 °C. Beads were then applied to MicroSpin columns (Pierce) and washed three times in TG lysis buffer and twice in wash buffer (50 mm Tris, pH 8.0). Protein was eluted in 30 μl of Laemmli sample buffer and processed for SDS-PAGE and Western blot.

##### Subcellular Fractionation

The membrane association assay was performed as described (27) with modifications. HEK 293 cells in 6-well dishes were transfected with 4 μg of FLAG-*Nmnat2* or variant construct, or co-transfected with 2 μg each of FLAG-*Nmnat2* and the relevant *zDHHC*-HA (or empty vector control) constructs. 24 h after transfection, cells were harvested in ice-cold DMEM, washed once in ice-cold PBS, and resuspended in 400 μl of buffer 1 (150 mm NaCl, 50 mm HEPES, pH 7.4, 24 μg/ml of digitonin (Sigma)). After a 10-min end-over-end mixing at 4 °C, samples were spun for 1 min at 5000 × *g* in a bench-top microcentrifuge at 4 °C and the supernatant was collected as the cytosolic fraction. The pellet was washed once in ice-cold PBS and resuspended in 400 μl of buffer 2 (150 mm NaCl, 50 mm HEPES, pH 7.4, 1% IGEPAL CA-630). Complete resuspension was ensured by vortexing samples briefly. After incubation on ice for 30 min, samples were spun for 1 min at 9,000 × *g* in a bench-top microcentrifuge at 4 °C and the supernatant was collected as the membrane/organelle fraction. All samples were diluted 1:1 into Laemmli SDS sample buffer and processed for Western blot. For each sample, the ratio of FLAG signal in the cytosolic *versus* membrane fractions was determined and normalized to control.

##### Protein Cross-linking

Twenty-four hours after transfection with 4 μg of FLAG-*Nmnat2*, HEK 293 cells in 6-well dishes were treated with 50 mm HA for 60 min or with 1 mm dithiobis(succinimidyl propionate) for 30 min, followed by 15 min quenching in 1 m Tris (pH 7.5) and processed for SDS-PAGE. For reducing conditions, sample buffer contained 5% β-mercaptoethanol and samples were heated to 95 °C for 5 min. For non-reducing conditions, β-mercaptoethanol was omitted from sample buffer and samples were heated to 80 °C for 3 min.

##### RNA Extraction and qRT-PCR

Total RNA was extracted from NSC34 cells using TriSure reagent (Bioline), followed by generation of cDNA using Superscript 2 reverse transcriptase (Invitrogen), according to the manufacturer's instructions. Quantitative RT (qRT)-PCR measurements of *zDHHC7* and *zDHHC17* mRNA abundance were performed on a Rotor-Gene Q Model 2-Plex HRM cycler running Rotor-Gene Q Software version 2.3.1 (Qiagen) and quantified using the Δ(Δ*C_t_*) method by reference to *Actb* and *Gusb*. The following primers were used: *zDHHC7* (forward, gagaaccatgctcactgacc; reverse, gcacttgtagatcacctcgc; expected amplicon mRNA: 103 bp, genomic: 1103 bp), *zDHHC 17* (forward, gtttcacttcctgtgggtgg; reverse, gcttgtatctcctggcgttc; expected amplicon mRNA: 103 bp, genomic: 707 bp), *Actb* (forward, tgaaccctaaggccaaccgt; reverse, aggcatacagggacagcaca; expected amplicon mRNA: 104 bp, genomic: 560 bp), *Gusb* (forward, gttgaggatcaacagtgccc; reverse, atgtcagcctcaaaggggag; expected amplicon size mRNA: 98 bp, genomic: 313 bp). The presence of a single amplicon in each reaction was confirmed by melt curve analysis and agarose gel electrophoresis after completion of the qRT-PCR cycle.

## RESULTS

### 

#### 

##### NMNAT2 Is a Substrate for Thioesterases APT1 and APT2

**FIGURE 1.**
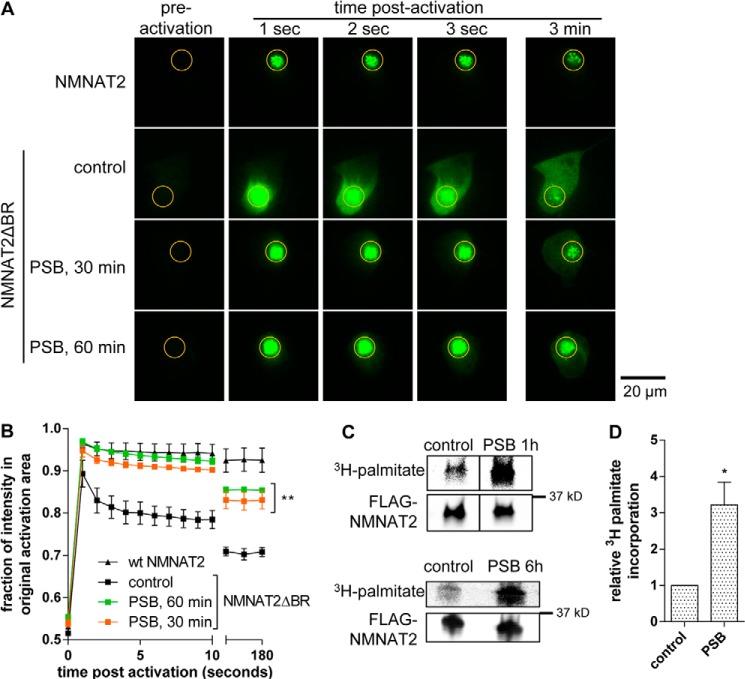
**Palmostatin B treatment boosts NMNAT2 membrane association and palmitoylation.** *A,* individual frames from photoactivation assay of SCG primary culture neurons expressing NMNAT2-PA-GFP or NMNAT2ΔBR-PA-GFP. APT inhibitor PSB (50 μm) or DMSO (control) were added to NMNAT2ΔBR-PA-GFP expressing cells 30 or 60 min prior to imaging. The region of activation is indicated by an *orange circle* in each image. *B,* quantification of protein mobility in *A. Error bars* indicate S.E., *n* = 3 independent experiments. Protein mobility of NMNAT2ΔBR was significantly decreased by addition of PSB (**, *p* < 0.001, non-linear curve fit). *C,* [^3^H]palmitate label and Western blots of FLAG-NMNAT2. HEK 293 cells expressing FLAG-NMNAT2 were labeled with [^3^H]palmitate with 50 μm PSB present (*PSB*) during the last hour of palmitate labeling (*top*) or throughout the labeling period (6 h, *bottom*) and subjected to FLAG-immunoprecipitation and processed for PhosphorImaging and Western blot. Note that an intervening band has been cropped out of the top blot (1 h PSB) but that brightness and contrast settings are identical between bands. *D,* quantification of palmitate incorporation. As there was no statistically significant difference between samples treated with PSB for 1 or 6 h, values were combined for analysis. Intensity of detected radiolabel was normalized to the FLAG signal on Western blot for each condition. For presentation, values were normalized to control. *Error bars* indicate S.E., *n* = 4 independent experiments. *, indicates statistically significant difference compared with control (*, *p* < 0.05; paired *t* test).

We previously reported that the cISTID of NMNAT2 is necessary and sufficient for membrane targeting and that, within the cISTID region, a double cysteine motif and several surrounding basic residues are necessary for efficient palmitoylation and membrane binding (11). Deletion of the entire cISTID region or specific mutation of the palmitoylated cysteine residues within the cISTID abolish NMNAT2 palmitoylation and result in a diffuse, cytosolic distribution of the mutant proteins (11). Photoactivation of fluorescently tagged NMNAT2 variants in SCG neurons (see “Experimental Procedures”) illustrates that wild-type NMNAT2 tagged to PA-GFP is strongly membrane bound, with most of the fluorescence signal remaining in the originally activated area of the cell body (Fig. 1, *A* and *B*). In contrast, NMNAT2ΔBR, in which basic residues surrounding the Cys^164–165^ palmitoylation site within the cISTID region are mutated (11), shows a much more diffuse localization, with most of the fluorescence signal spreading throughout the cell body and only a small fraction remaining within the originally activated area (Fig. 1, *A* and *B*; see Table 1 for an overview of the properties of all NMNAT2 mutant constructs used). Importantly, NMNAT2ΔBR is still palmitoylated (albeit to a much lesser extent than wild-type NMNAT2) and, like wild-type NMNAT2, a portion of it is targeted to Golgi membranes in the cell body and Golgi-derived axonal transport vesicles (although a partial association with the ER in cell bodies cannot be excluded) (11). The strong reduction in membrane association relative to wild-type NMNAT2 means that NMNAT2ΔBR is a useful tool to study potential increases in NMNAT2 membrane binding.

To test whether NMNAT2 can undergo depalmitoylation mediated by the cytosolic thioesterases APT1 or APT2, we used PSB, an inhibitor of APT1 and APT2 thioesterase activity (23, 28). PSB (50 μm) was applied to SCG neurons for 30 or 60 min prior to imaging and led to a significant increase in membrane association of NMNAT2ΔBR-PA-GFP (Fig. 1, *A* and *B*). This suggests that a PSB-sensitive thioesterase activity limits the extent of NMNAT2ΔBR membrane association and that depalmitoylation by thioesterases is a necessary step for the release of NMNAT2 from membranes. The fact that PSB treatment affected the subcellular localization of NMNAT2ΔBR on relatively short time scales suggests that depalmitoylation could influence the localization and turnover of endogenous NMNAT2, which has a half-life of less than 1 h (11). Using [^3^H]palmitate labeling in HEK 293 cells, we found that PSB treatment significantly boosted the steady-state incorporation of labeled palmitate into FLAG-NMNAT2, independently of whether it was applied for the whole labeling period or only during the last hour of [^3^H]palmitate labeling (Fig. 1, *C* and *D*). Although technical constraints mean that we were not able to directly measure the rate of [^3^H]palmitate incorporation into NMNAT2 in SCG neurons, our findings nevertheless, indicate that the effects of PSB treatment are qualitatively similar in both cell types, and that the above effects of PSB on membrane association are mediated through effects on NMNAT2 palmitoylation.

**FIGURE 2.**
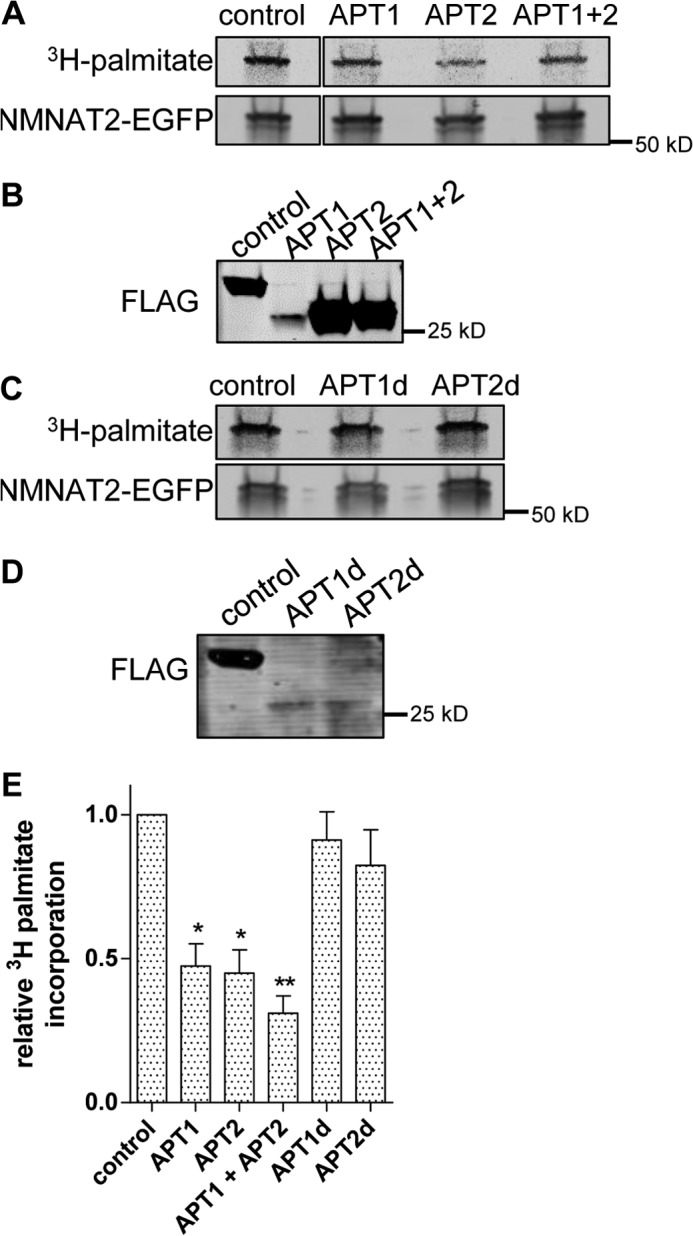
**APT1 and APT2 overexpression reduce NMNAT2 palmitoylation.** *A,* [^3^H]palmitate labeling and Western blot of NMNAT2-EGFP. HEK 293 cells expressing NMNAT2-EGFP together with FLAG-NMNAT1 (*control*), FLAG-APT1 (*APT1*), FLAG-APT2 (*APT2*), or FLAG-APT1 and FLAG-APT2 (*APT1* + *2*) were labeled with [^3^H]palmitate, subjected to EGFP immunoprecipitation and processed for PhosphorImaging and Western blot. All images are of the same gel with the same corrections (brightness and contrast) applied but with an intervening lane cropped out. *B,* input samples from *A* showing expression of FLAG-NMNAT1 (control), FLAG-APT1, and/or FLAG-APT2 as indicated. *C,* [^3^H]palmitate labeling and Western blot of NMNAT2-EGFP. HEK 293 cells expressing NMNAT2-EGFP together with FLAG-NMNAT1 (control), enzyme-dead FLAG-APT1^S119A^ (*APT1d*), or enzyme-dead FLAG-APT2^S122A^ (*APT2d*) were labeled with [^3^H]palmitate, subjected to EGFP immunoprecipitation, and processed for PhosphorImaging and Western blot. *D,* input samples from *C* showing expression of FLAG-NMNAT1 (*control*), FLAG-APT1^S119A^ (*APT1d*), or FLAG-APT2^S122A^ (*APT2d*) as indicated. *E,* quantification of palmitate incorporation. Intensity of detected radiolabel was normalized to EGFP signal on Western blot for each condition. For presentation, values were normalized to control. *Error bars* indicate S.E., *n* = 3 independent experiments. *, indicates statistically significant difference compared with control (*, *p* < 0.05; **, *p* < 0.01; one-way analysis of variance with Tukey's Multiple Comparisons post-test).

Next, we sought to confirm that the effects of PSB treatment on NMNAT2 palmitoylation were indeed mediated by APT1 and/or APT2. Co-overexpression of NMNAT2-EGFP with FLAG-APT1 and/or FLAG-APT2 significantly reduced the incorporation of [^3^H]palmitate into NMNAT2-EGFP to similar extents (Fig. 2, *A* and *E*), despite the observation that FLAG-APT2 was expressed at significantly higher levels than FLAG-APT1 in this assay (Fig. 2*B*). To test whether the thioesterase activities of APT1 and APT2 were required for their effect on [^3^H]palmitate incorporation into NMNAT2, we created enzyme-dead variants of both thioesterases based on previously published findings (29, 30). Co-overexpression of FLAG-APT1^S119A^ or FLAG-APT2^S122A^ did not alter palmitate incorporation into NMNAT2-EGFP (Fig. 2,*C* and *E*). However, it is worth noting that the enzyme-dead variant of FLAG-APT2 was expressed at significantly lower levels than the wild-type in this assay (Fig. 2*D*). Nevertheless, these results suggest that APT1 and APT2 are both able to efficiently depalmitoylate NMNAT2 under these conditions and that their thioesterase activities are required for this effect.

##### Depalmitoylation Is Insufficient for NMNAT2 Membrane Detachment

**FIGURE 3.**
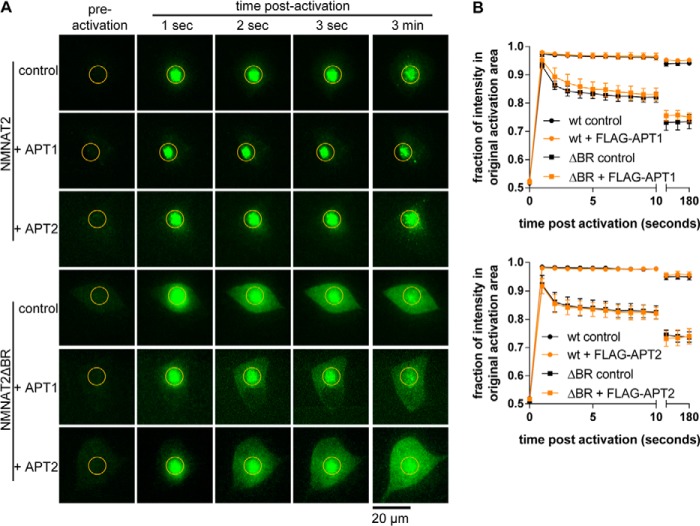
**Overexpression of APT1 or APT2 does not release NMNAT2 from membranes.** *A,* individual frames from photoactivation assay of SCG primary culture neurons expressing NMNAT2-PA-GFP or NMNAT2ΔBR-PA-GFP together with FLAG-NMNAT1 (*control*), FLAG-APT1 or FLAG-APT2. The region of activation is indicated by an *orange circle* in each image. *B,* quantification of protein mobility in *A. Error bars* indicate S.E., *n* = 3 independent experiments. Protein mobility of NMNAT2-PA-GFP or NMNAT2ΔBR-PA-GFP was not altered significantly by presence of FLAG-APT1 or FLAG-APT2 (*p* > 0.05; non-linear curve fit).

**FIGURE 4.**
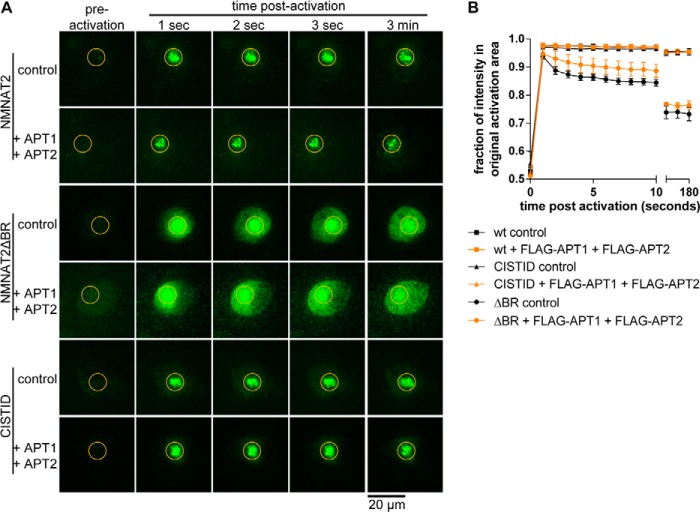
**Combined overexpression of APT1 and APT2 does not release NMNAT2 from membranes.** *A,* individual frames from photoactivation assay of SCG primary culture neurons expressing NMNAT2-PA-GFP, NMNAT2ΔBR-PA-GFP, or cISTID-PA-GFP together with FLAG-NMNAT1 (*control*) or FLAG-APT1 and FLAG-APT2. The region of activation is indicated by an *orange circle* in each image. *B,* quantification of protein mobility in *A. Error bars* indicate S.E., *n* = 3 independent experiments. Protein mobility was not altered significantly by the presence of FLAG-APT1 and FLAG-APT2 in any of the conditions (*p* > 0.05; non-linear curve fit).

Based on the above results, we hypothesized that depalmitoylation of NMNAT2 by APT1 and APT2 could release NMNAT2 from membranes. To test this idea, we co-overexpressed NMNAT2-PA-GFP or NMNAT2ΔBR-PA-GFP together with FLAG-APT1 or FLAG-APT2. Interestingly, neither APT1 nor APT2 were able to release NMNAT2 from membranes (Fig. 3). Moreover, even combined overexpression of FLAG-APT1 and FLAG-APT2 had no effect on the membrane retention of NMNAT2 or NMNAT2ΔBR (Fig. 4). Given that overexpression of APT1 and APT2 only reduced [^3^H]palmitate incorporation into NMNAT2 by around 60% (Fig. 2*E*), it is possible that more complete depalmitoylation by APT1 and APT2, or by other thioesterases, is required to successfully release NMNAT2 from membranes. However, an interesting alternative is the possibility that depalmitoylation is not sufficient to release NMNAT2 from membranes and that other mechanisms could maintain stable anchoring of NMNAT2. Thus, we aimed to define the sequence requirements of NMNAT2 for this potential post-palmitoylation membrane anchoring mechanism.

We found that a cISTID-PA-GFP construct, which we previously showed to be necessary and sufficient for NMNAT2 membrane targeting (11) was also retained at membranes in the presence of APT1 and APT2 (Fig. 4). This suggests that the putative alternative mechanism of membrane association that allows NMNAT2 to remain membrane bound in the presence of overexpressed APT1 and APT2 is mediated by sequences within the cISTID region, but does not require the basic residues mutated in NMNAT2ΔBR. Together, our data suggest that boosting levels of NMNAT2 palmitoylation through PSB treatment can drive more of the protein to membranes (Fig. 1), but a decrease of palmitoylation levels by overexpression of APT1 and/or APT2 is not sufficient to increase its rate of membrane dissociation (Figs. 3 and 4).

**FIGURE 5.**
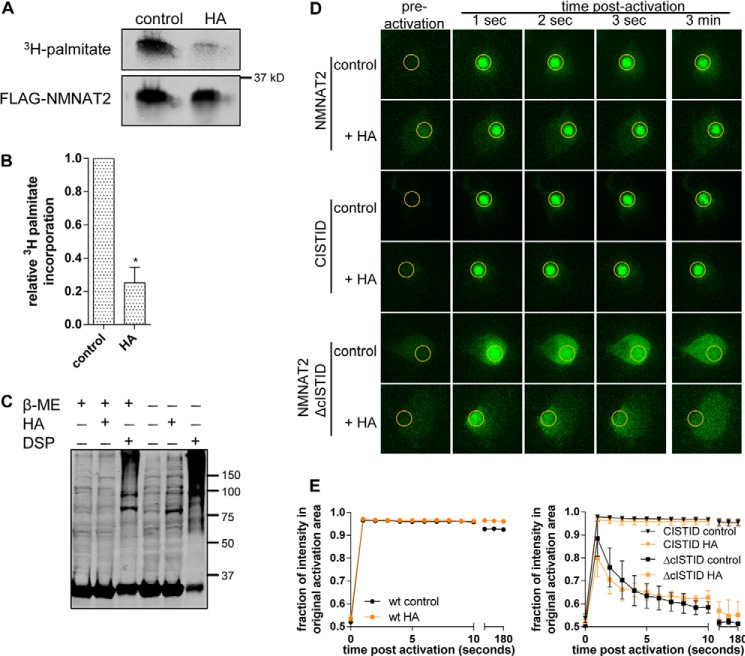
**Chemical depalmitoylation does not release NMNAT2 from membranes.** *A,* [^3^H]palmitate labeling and Western blot of FLAG-NMNAT2. HEK 293 cells expressing FLAG-NMNAT2 were labeled with [^3^H]palmitate for a total of 6 h, in the absence (*control*) or presence (*HA*) of 50 mm hydroxylamine (*HA*) during the final hour of incubation. Following lysis, samples were subjected to FLAG immunoprecipitation and processed for PhosphorImaging and Western blot. *B,* quantification of palmitate incorporation. Intensity of the detected radiolabel was normalized to the FLAG signal on Western blots for each condition. For presentation, values of the HA-treated samples was normalized to control. *Error bars* indicate S.E., *n* = 3 independent experiments. *, indicates statistically significant difference compared with control (*, *p* < 0.05; *t* test). *C,* Western blot of FLAG-NMNAT2. HEK 293 cells expressing FLAG-NMNAT2 were treated with 50 mm HA for 1 h or with 1 mm dithiobis(succinimidyl propionate) for 30 min and separated by reducing (5% β-mercaptoethanol, β-*ME*) or non-reducing SDS-PAGE. *D,* individual frames from photoactivation assay of SCG primary culture neurons expressing NMNAT2-PA-GFP (*top*) or cISTID-PA-GFP (*bottom*) after incubation for 1 h in the absence (*control*) or presence (*HA*) of 50 mm HA. The region of activation is indicated by an *orange circle* in each image. *E,* quantification of protein mobility in the presence and absence of HA. *Error bars* indicate S.E., *n* = 3–4 independent experiments. Protein mobility of NMNAT2-PA-GFP, cISTID-PA-GFP, or NMNAT2ΔcISTID-PA-GFP was not altered significantly by treatment with HA (*p* > 0.05; non-linear curve fit).

To further confirm that NMNAT2 can remain membrane associated following depalmitoylation, we used the thiol-reactive compound HA for chemical depalmitoylation. HA treatment (50 mm, 60 min) significantly reduced the incorporation of [^3^H]palmitate into FLAG-NMNAT2 in HEK 293 cells (Fig. 5, *A* and *B*). The same treatment, however, did not alter the membrane association status of NMNAT2-PA-GFP or cISTID-PA-GFP (Fig. 5, *D* and *E*). To account for potential off-target effects of HA on cells in this assay, we confirmed that HA treatment did not induce detectable protein cross-linking under these conditions, whereas dithiobis(succinimidyl propionate), a known cross-linking agent containing a disulfide bond, caused a significant accumulation of high molecular weight species that was sensitive to reducing conditions on SDS-PAGE (Fig. 5*C*). Moreover, we excluded a nonspecific effect of HA treatment on protein mobility by confirming that the mobility of the cytosolic NMNAT2ΔcISTID mutant was not affected by HA exposure (Fig. 5, *D* and *E*). Together, these data suggest the existence of a palmitoylation-independent mechanism of stable membrane association for NMNAT2 mediated by the cISTID region.

##### Depalmitoylation Does Not Affect NMNAT2 Ubiquitination and Turnover

**FIGURE 6.**
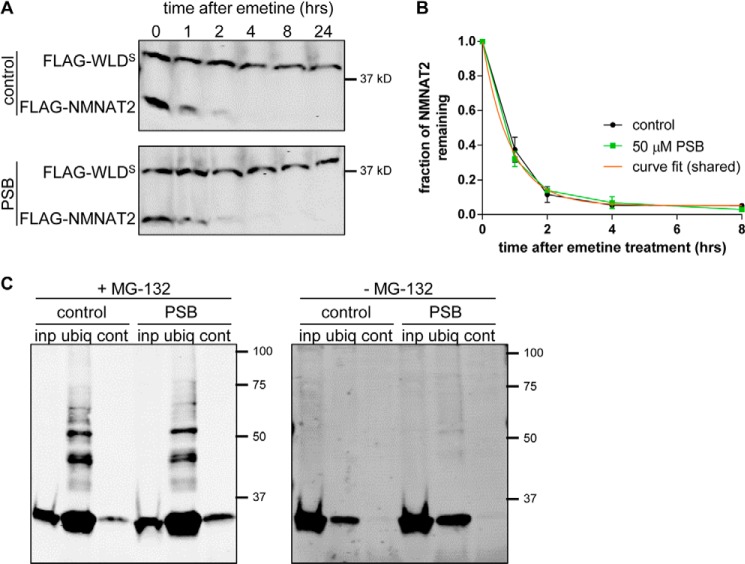
**Palmostatin B treatment does not affect NMNAT2 turnover or ubiquitination.** *A,* representative Western blots of HEK 293 cells co-transfected with FLAG-*Wld^S^* and FLAG-*Nmnat2* and maintained in the presence (PSB, 50 μm) or absence (control) of PSB. 24 h after transfection, cells were treated with 10 μm emetine for the amount of time indicated after which samples were processed for SDS-PAGE and Western blot using anti-FLAG antibody. *B*, quantification of NMNAT2 turnover after emetine treatment in the presence or absence of PSB. For each sample and time point the amount of FLAG-NMNAT2 remaining was normalized to FLAG-WLD^S^ as an internal control. *Error bars* indicate S.E., *n* = 3 independent experiments. Half-lives were not significantly different (*p* = 0.66; non-linear curve fit) and the *orange line* indicates the shared, fitted exponential decay curve. *C*, representative Western blots of GST-DSK2 pulldown assay. HEK 293 cells expressing FLAG-NMNAT2 were maintained in the presence (PSB, 50 μm) or absence (control) of PSB. Six hours prior to lysis, fresh medium containing MG-132 (*left*) or control medium without MG-132 (*right*) was added. Cells were lysed (*inp*., total input) and ubiquitinated proteins were immunoprecipitated using GST-DSK2 bound to glutathione beads (*ubiq*.). GST-fused mutant DSK2 was used for control pulldown (*cont*.). Eluted proteins were processed for SDS-PAGE and analyzed by Western blot using anti-FLAG antibody.

Given our previous results regarding the localization dependence of NMNAT2 ubiquitination and turnover (11), we thought it interesting to ask whether the rate of NMNAT2 depalmitoylation by APT1 and APT2 affects its ubiquitination or turnover. To this end, we performed emetine chase experiments in HEK 293 cells to determine the rate of turnover of FLAG-NMNAT2. Interestingly, we found that inhibition of APT1 and APT2 with PSB did not affect the rate of NMNAT2 turnover in these cells (Fig. 6, *A* and *B*). Similarly, we found no evidence for altered levels of ubiquitination of FLAG-NMNAT2 after PSB treatment in ubiquitin pull-down experiments (Fig. 6*C*). This observation is consistent with the above findings indicating a dissociation between NMNAT2 depalmitoylation and membrane dissociation as our previous results suggested that membrane association, rather than palmitoylation status, determines NMNAT2 ubiquitination and its rate of turnover (11).

##### A Subset of zDHHC Proteins Are Candidate Palmitoyltransferases for NMNAT2

**FIGURE 7.**
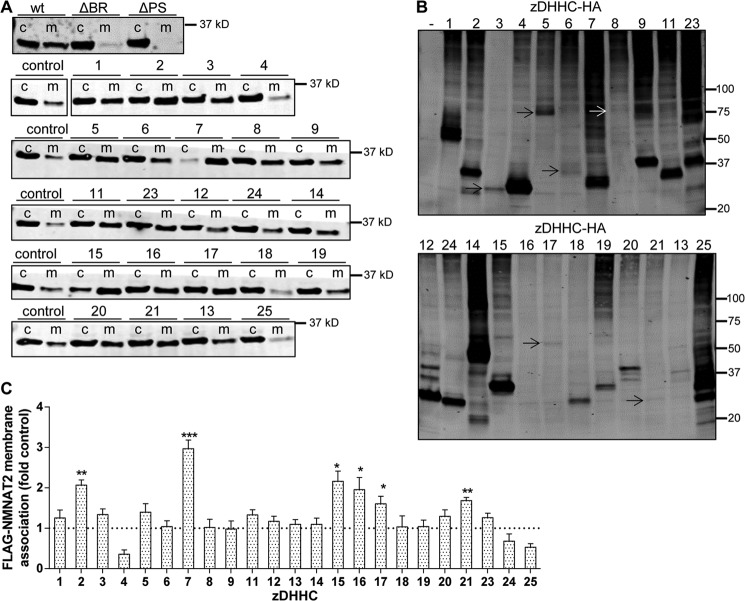
**Candidate zDHHC palmitoyltransferases increase NMNAT2 membrane association.** *A,* representative Western blots of NMNAT2 membrane association assay. HEK 293 cells expressing FLAG-NMNAT2 (*wt*), FLAG-NMNAT2ΔBR (Δ*BR*), FLAG-NMNAT2ΔPS (Δ*PS*), or co-expressing FLAG-NMNAT2 and empty vector (*control*) or one of 23 zDHHC-HA constructs were subjected to a simple subcellular fractionation protocol to separate cytosolic (*c*) and membrane (*m*) fractions and processed for SDS-PAGE and Western blot using anti-FLAG antibody. Note that an intervening lane has been cropped out in the *second from top blot. B,* Western blot of input samples from *A* showing expression of HA-tagged zDHHCs. Where only faint bands are visible due to exposure settings, these are indicated by *arrows. C,* quantification of FLAG-NMNAT2 membrane-to-cytosol ratio in presence of empty vector (*control*) or expression of one of the zDHHC-HA enzymes. For presentation, values were normalized to control. *Error bars* indicate S.E., *n* = 4 independent experiments. *, indicates statistically significant difference compared with control (*, *p* < 0.05; **, *p* < 0.01; ***, *p* < 0.001; one-way analysis of variance with Tukey's multiple comparisons post-test).

Next, we aimed to identify palmitoyltransferase enzymes that could mediate NMNAT2 palmitoylation. In an initial screen, we assayed the ability of 23 mouse zDHHC family palmitoyltransferases to boost membrane targeting of FLAG-NMNAT2 when co-overexpressed in HEK 293 cells using a subcellular fractionation method previously used to identify candidate zDHHC enzymes for other substrates (31, 32). We identified six candidate zDHHC enzymes that significantly increased NMNAT2 membrane association in this assay (Fig. 7, *A* and *C*). It is important to note that expression levels of individual zDHHC enzymes in this assay varied considerably (Fig. 7*B*). However, there was no correlation between the expression levels of individual zDHHC enzymes and their ability to increase membrane association of NMNAT2 in this assay (*e.g.* compare zDHHC 17 and zDHHC9 in Fig. 7, *B* and *C*). Interestingly, a few of the zDHHC enzymes appeared to reduce NMNAT2 membrane association. This observation is consistent with previous studies using this technique (31) but the underlying mechanism of this suppression of membrane association is currently unknown. However, zDHHC 13 and zDHHC25 were previously shown to suppress incorporation of [^3^H]palmitate into PDE10A (33) so the reduction in NMNAT2 membrane association by zDHHC4 and zDHHC25 observed here could indeed represent a reduction in NMNAT2 palmitoylation.

**FIGURE 8.**
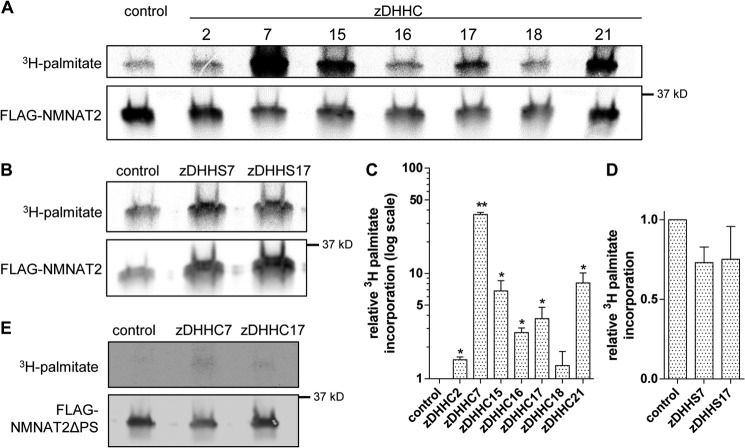
**Overexpression of candidate zDHHC enzymes boosts NMNAT2 palmitoylation.** *A* and *B,* [^3^H]palmitate labeling and Western blot of FLAG-NMNAT2. HEK 293 cells expressing FLAG-NMNAT2 only (*control*) or FLAG-NMNAT2 together with the indicated zDHHC-HA (*A*) or an enzyme-dead zDHHS-HA mutant (*B*) were labeled with [^3^H]palmitate, subjected to FLAG immunoprecipitation, and processed for PhosphorImaging and Western blot. *C* and *D,* quantification of palmitate incorporation in *A* and *B*, respectively. Intensity of detected radiolabel was normalized to FLAG signal on Western blot for each condition. For presentation, values were normalized to control. *Error bars* indicate S.E., *n* = 3 independent experiments. *, indicate statistically significant difference compared with control (*, *p* < 0.05; **, *p* < 0.01; ***, *p* < 0.001; one-way analysis of variance with Tukey's multiple comparisons post-test). *E,* [^3^H]palmitate labeling and Western blot of FLAG-NMNAT2ΔPS. HEK 293 cells expressing FLAG-NMNAT2 ΔPS only (*control*) or FLAG-NMNAT2 together with zDHHC7 or zDHHC17 were labeled with [^3^H]palmitate, subjected to FLAG immunoprecipitation, and processed for PhosphorImaging and Western blot analysis.

To confirm that the observed increases in NMNAT2 membrane association in the presence of candidate zDHHC enzymes were due to increased levels of NMNAT2 palmitoylation, we performed [^3^H]palmitate labeling of FLAG-NMNAT2 in HEK 293 cells co-overexpressing individual zDHHC enzymes. As predicted, incorporation of [^3^H]palmitate into FLAG-NMNAT2 was significantly increased by all six candidate zDHHC enzymes (Fig. 8, *A* and *C*) but not by a zDHHC enzyme that did not alter membrane association of NMNAT2 in the subcellular fractionation assay above (zDHHC18).

**FIGURE 9.**
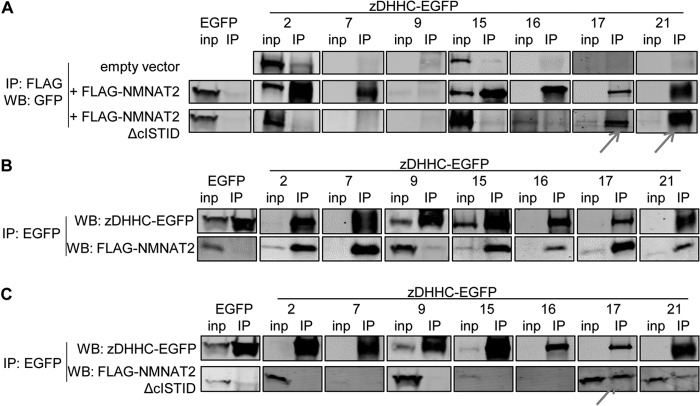
**Co-immunoprecipitation of NMNAT2 and candidate zDHHC palmitoyltransferases.** *A,* representative Western blots of zDHHC-EGFP palmitoyltransferases co-immunoprecipitated with wild-type or mutant FLAG-NMNAT2. HEK 293 cells expressing candidate zDHHC-EGFP constructs together with an empty vector control (*top row*), FLAG-NMNAT2 (*middle row*) or mutant FLAG-NMNAT2ΔcISTID (*bottom row*) were lysed (*inp*, total input), subjected to immunoprecipitation using anti-FLAG antibody (*IP*), and processed for SDS-PAGE and Western blot (*WB*) analysis using anti-GFP antibody. *B* and *C,* representative Western blots of wild-type or mutant FLAG-NMNAT2 co-immunoprecipitated with zDHHC-EGFP palmitoyltransferases. HEK 293 cells expressing FLAG-NMNAT2 (*B*) or FLAG-NMNAT2ΔcISTID (*C*) together with untagged EGFP control or candidate zDHHC-EGFP constructs were lysed (*inp*, total input), subjected to immunoprecipitation using anti-GFP antibody (*IP*) and processed for SDS-PAGE and Western blot using anti-GFP and anti-FLAG antibodies. Apparent molecular masses of tagged proteins on Western blots were as follows: FLAG-NMNAT2, 34 kDa; FLAG-NMNAT2ΔcISTID, 30 kDa; EGFP, 26 kDa; zDHHC2-EGFP, 65 kDa; zDHHC7-EGFP, 50 kDa; zDHHC9-EGFP, 65 kDa; zDHHC15-EGFP, 68 kDa; zDHHC16-EGFP, 68 kDa; zDHHC17-EGFP, 100 kDa; zDHHC21-EGFP, 45 kDa.

To further support an enzyme-substrate interaction between the candidate zDHHC enzymes and NMNAT2, we performed co-immunoprecipitation assays as enzyme-substrate complexes have been demonstrated for several zDHHC-substrate pairings (34–36). We found that all candidate zDHHC-EGFP constructs, but not EGFP alone, or zDHHC9, which did not alter NMNAT2 membrane association, were significantly enriched in FLAG immunoprecipitation in the presence of FLAG-NMNAT2 but not in its absence (Fig. 9*A*). Conversely, FLAG-NMNAT2 was significantly co-immunoprecipitated by all zDHHC candidates, but not by EGFP alone or by zDHHC9 (Fig. 9*B*). Although expression levels vary considerably between zDHHC enzymes in this assay (see Fig. 7*B*), there is no correlation between the relative expression levels of zDHHC enzymes and the extent of their co-immunoprecipitation with NMNAT2, suggesting that these are specific, expression level independent effects. For interaction with most candidate zDHHC enzymes, the cISTID region of NMNAT2 appears to be required, as a cISTID-deficient FLAG-NMNAT2ΔcISTID did not co-immunoprecipitate candidate zDHHC enzymes (Fig. 9*A*) and was not co-immunoprecipitated by candidate zDHHC enzymes (Fig. 9*C*). Interestingly, however, zDHHC17 and zDHHC21 appear to be an exception to this, as we observed a bidirectional co-immunoprecipitation of these zDHHCs and FLAG-NMNAT2ΔcISTID (Fig. 9, *A* and *C*, *arrows*). This suggests that the interactions between NMNAT2 and zDHHC17 or zDHHC21 are partially mediated by regions outside of the minimum cISTID region of NMNAT2 that is required for palmitoylation. For zDHHC17, this observation is in agreement with reports for other substrates for which sequences outside of the minimum palmitoylation domain of the substrate have been shown to interact with the zDHHC17 palmitoyltransferase (34, 37).

**FIGURE 10.**
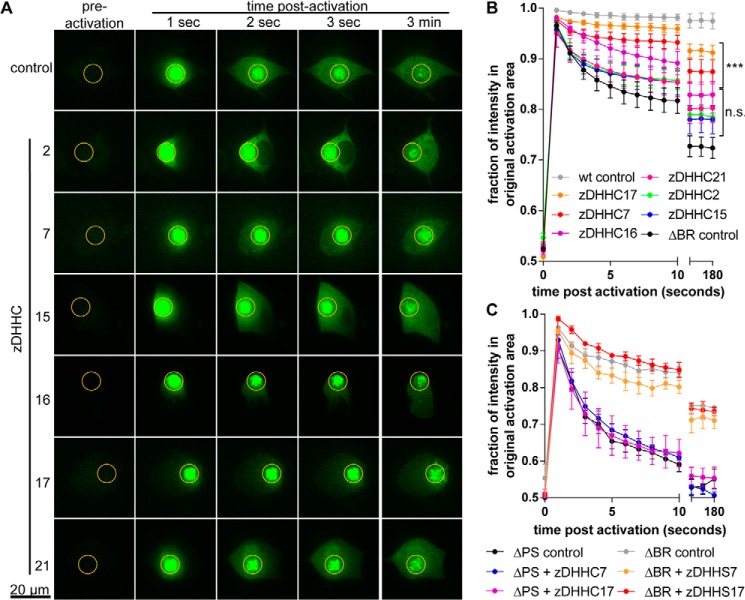
**Overexpression of candidate zDHHC palmitoyltransferases increases NMNAT2 membrane association.** *A,* individual frames from photoactivation assay of SCG primary culture neurons expressing NMNAT2ΔBR-PA-GFP or co-expressing NMNAT2ΔBR-PA-GFP and candidate zDHHC-HA constructs. The region of activation is indicated by an *orange circle* in each image. *B,* quantification of protein mobility in *A. Error bars* indicate S.E., *n* = 3 independent experiments. *, indicates statistically significant difference compared with NMNAT2ΔBR-PA-GFP control (*n.s*., non-significant; ***, *p* < 0.001; non-linear curve fit). *C,* quantification of protein mobility in photoactivation assay. Mobility of NMNAT2ΔBR-PA-GFP was not altered significantly by co-expression of enzyme-dead zDHHS7 or zDHHS17 and mobility of NMNAT2ΔPS-PA-GFP was not affected by co-expression of zDHHC7 or zDHHC17. *Error bars* indicate S.E., *n* = 3 independent experiments.

To confirm the observations from the membrane association assay in neuronal cells, we determined the membrane association of NMNAT2ΔBR in the presence of individual candidate zDHHC enzymes using the photoactivation assay in SCG cell bodies. Co-expression of zDHHC2, zDHHC15, zDHHC16, and zDHHC21 had modest, statistically non-significant effects on the membrane association of NMNAT2ΔBR-PA-GFP (Fig. 10, *A* and *B*). In contrast, zDHHC7 and zDHHC17 both led to statistically significant increases in membrane retention of NMNAT2ΔBR-PA-GFP (Fig. 10, *A* and *B*). These findings suggest zDHHC7 and zDHHC17 as the strongest candidates for zDHHC palmitoyltransferases that affect the subcellular localization of NMNAT2 in neurons.

Given the potential roles of zDHHC7 and zDHHC17 in mediating NMNAT2 palmitoylation, we sought to confirm that their observed effects on NMNAT2 membrane association and palmitoylation were dependent on their enzyme activities. We found that enzyme-dead zDHHC7^C160S^ (referred to as zDHHS7) and zDHHC17^C467S^ (zDHHS17) did not promote [^3^H]palmitate incorporation into FLAG-NMNAT2 (Fig. 8, *B* and *D*). Accordingly, overexpression of zDHHS7 or zDHHS17 did not affect the membrane targeting of NMNAT2ΔBR in the photoactivation assay (Fig. 10*C*). Moreover, we sought to confirm the requirement for the Cys^164^/Cys^165^ palmitoylation site in NMNAT2 by demonstrating that overexpression of zDHHC7 or zDHHC17 did not lead to an increase in [^3^H]palmitate incorporation into NMNAT2ΔPS (Fig. 8*E*), or to an increase in its membrane association in the photoactivation assay (Fig. 10*C*). Together, these results indicate that zDHHC7 and zDHHC17 affect NMNAT2 palmitoylation and subcellular localization through mechanisms that require intact palmitoylation sites in both the enzymes and the substrate.

##### Endogenous zDHHC17 Affects NMNAT2 Palmitoylation

**FIGURE 11.**
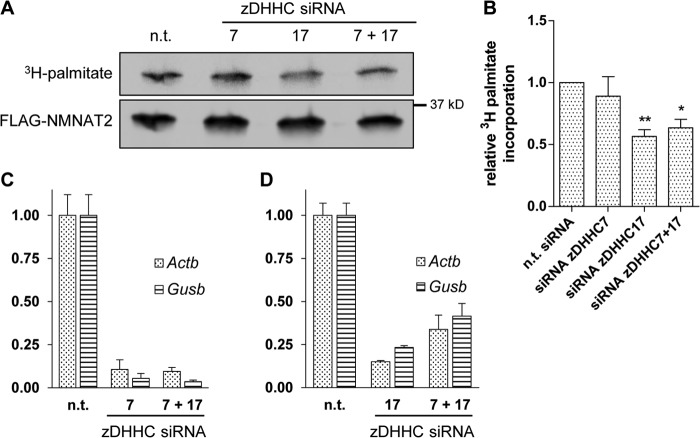
**Endogenous zDHHC17 contributes to NMNAT2 palmitoylation in NSC34 cells.** *A*, [^3^H]palmitate labeling and Western blot of FLAG-NMNAT2. NSC34 cells transfected with FLAG-*Nmnat2* together with non-targeting siRNA control (*n.t*.) or siRNA against *zDHHC7* and/or *zDHHC17* as indicated were labeled with [^3^H]palmitate, subjected to FLAG-immunoprecipitation, and processed for PhosphorImaging and Western blot. *B*, quantification of palmitate incorporation. Intensity of detected radiolabel was normalized to the FLAG signal on Western blot for each condition. For presentation, values were normalized to control. *Error bars* indicate S.E., *n* = 4 independent experiments. *, indicates statistically significant difference compared with control (*, *p* < 0.05; **, *p* < 0.01; one-way analysis of variance with Tukey's multiple comparisons post-test). *C,* quantification of siRNA knockdown efficiency of *zDHHC7* mRNA. qRT-PCR measurements of *zDHHC7* mRNA abundance were quantified relative to *Actb* and *Gusb* and normalized to control (*n.t*., non-targeting siRNA control). *Error bars* indicate S.E., *n* = 3 independent experiments. *D*, quantification of siRNA knockdown efficiency of *zDHHC17* mRNA. qRT-PCR measurements of *zDHHC17* mRNA abundance were quantified relative to *Actb* and *Gusb* and normalized to control (*n.t*., non-targeting siRNA control). *Error bars* indicate S.E., *n* = 3 independent experiments.

Although the above results establish zDHHC7 and zDHHC17 as candidate zDHHC palmitoyltransferases with an influence on NMNAT2 subcellular localization in neurons, they were based on overexpression of both NMNAT2 and the zDHHC enzyme. To test whether endogenous zDHHC7 or zDHHC17 are involved in NMNAT2 palmitoylation, we utilized an siRNA approach in the neuronal NSC34 cell line. We used qRT-PCR to confirm mRNA level expressions of *zDHHC7* and *zDHHC17* in this cell line and to test the efficiency of knock-down achieved by the siRNA. We found that both zDHHC candidates were expressed and that their mRNA expression levels were incompletely but significantly attenuated by the relevant siRNA treatment. Knockdown of *zDHHC7* was slightly more efficient than knockdown of *zDHHC17*, especially when both siRNAs were used in combination (Fig. 11, *C* and *D*). Although siRNA-mediated knockdown of zDHHC7 did not significantly affect [^3^H]palmitate incorporation into FLAG-NMNAT2 at 3 days post-transfection. In contrast, knockdown of zDHHC17, even though less complete than that of zDHHC7 at mRNA level, reduced [^3^H]palmitate labeling of FLAG-NMNAT2 by 40%, regardless of whether zDHHC7 was knocked down at the same time or not (Fig. 11, *A* and *B*). The observed incomplete suppression of [^3^H]palmitate incorporation could be the result of incomplete knockdown of zDHHC17 itself (see Fig. 11*D*) and/or the activity of alternative zDHHC enzymes partially compensating for the reduction in zDHHC17 levels. Nevertheless, our results strongly suggest a role for endogenous zDHHC17 in NMNAT2 palmitoylation in these cells.

## DISCUSSION

In this study, we identify a set of palmitoyltransferase and thioesterase enzymes that appear to regulate NMNAT2 palmitoylation levels and its subcellular localization. Interestingly, however, there appears to be some dissociation between palmitoylation and membrane attachment, suggesting that palmitoylation could play roles beyond the regulation of NMNAT2 subcellular localization.

Whereas previous studies reported substrate specificity in the depalmitoylation of target proteins by either APT1 (20, 38) or APT2 (21), NMNAT2 appears to be equally susceptible to depalmitoylation by both of these thioesterases. Similarly, a semi-synthetic N-Ras protein was also depalmitoylated by both APT1 and APT2 *in vitro* (28), but, to our knowledge, our results are the first suggestion of a protein substrate for both APT1 and APT2 in living cells.

Our results indicate that NMNAT2 membrane association can be maintained following depalmitoylation. However, for NMNAT2 to eventually dissociate from membranes, depalmitoylation likely remains a necessary step as inhibition of thioesterase activity by PSB led to increased membrane association of NMNAT2ΔBR. The observation that membrane association of NMNAT2 is maintained following depalmitoylation is in agreement with findings for other palmitoylated proteins including GAP-43 (39) and SNAP-25 (40). For NMNAT2, the cISTID region is sufficient to mediate this post-palmitoylation membrane attachment but basic residues within this region are not required. Thus, this mechanism of membrane association appears to be different from the initial membrane targeting mediated by cISTID basic residues that is necessary for efficient palmitoylation to occur in the first place (11). Although the precise mechanism behind this continued membrane association of depalmitoylated NMNAT2 remains to be elucidated, it is interesting to note that it requires palmitoylation to be established first, suggesting a multistep process of membrane targeting and stable association.

It is interesting to note that the set of zDHHC enzymes we identified as candidates for mediating palmitoylation of NMNAT2 (zDHHC2, -7, -15, -16, -17, and -21) shows extensive overlap with the series of zDHHC palmitoyltransferases found to promote palmitoylation of SCG10/stathmin2 (zDHHC2, -3, -7, -15, -17, and -21) (36). Both, NMNAT2 and SCG10, are labile, palmitoylated proteins that play important roles in axon survival and in determining the duration of the latent phase of Wallerian degeneration (1, 2, 41), so it is intriguing to speculate that their regulation by a similar group of zDHHC palmitoyltransferases could be functionally important for axon survival and the course of Wallerian degeneration.

The evidence presented here suggests that endogenous zDHHC17 is involved in the palmitoylation of NMNAT2. Using overexpression and knockdown approaches, our findings indicate that modulation of zDHHC17 levels is sufficient to alter the extent of NMNAT2 palmitoylation as well as its subcellular localization. zDHHC17 was found to palmitoylate a variety of target proteins (42), including huntingtin (43). Moreover, palmitoylation of a zDHHC17 target protein, as well as palmitoylation of zDHHC17 itself, were found to be impaired in a mouse model of Huntington disease (44) and loss of zDHHC17 in mice produces a phenotype that recapitulates several features of Huntington disease (44, 45), indicating that a failure to maintain proper palmitoylation dynamics of zDHHC17 target proteins could be one of the pathological features in Huntington disease. Given the role of NMNAT2 in axon survival (1, 2), the finding that zDHHC17 could regulate NMNAT2 palmitoylation suggests a potential impairment of NMNAT2 palmitoylation and subcellular targeting in Huntington disease models that warrants further investigation.

Further work is required to elucidate the functional effects of each of the enzymes identified here in regulating NMNAT2-mediated axon survival. However, given previous findings regarding the role of NMNAT2 subcellular localization and the duration of axon survival after axotomy, targeting the enzymes identified here might provide novel avenues to delay axon degeneration through modulation of NMNAT2 palmitoylation and subcellular localization.

## Footnotes

2 The abbreviations used are:

NMNAT2: nicotinamide mononucleotide adenylyltransferase 2
APT: acyl-protein thioesterase
cISTID: central isoform-specific targeting and interaction domain of NMNAT2
HA: hydroxylamine
PA-GFP: photoactivatable-green fluorescent protein
PSB: palmostatin B
SCG: superior cervical ganglion
qRT: quantitative reverse transcription.
